# Case report and literature review of stent detachment after vertebral artery stenting

**DOI:** 10.3389/fcvm.2026.1842580

**Published:** 2026-09-23

**Authors:** Qiangguo Wang, Feng Sun, Qian Liu, Zhenhui Du, Zhihai Ma, Zongbao Liu

**Affiliations:** 1 First Clinical Medical College, Gansu University of Chinese Medicine, Lanzhou, China; 2 Department of Cerebrovascular Diseases, Gansu Provincial Hospital, Lanzhou, China

**Keywords:** conservative treatment, posterior circulation ischemia, stent migration, stenting, vertebral artery stenosis

## Abstract

This study presents a case of severe stenosis at the origin of the left vertebral artery, which was complicated by stent detachment after stent implantation. We carried out a retrospective analysis of the patient's clinical data and undertook a review of relevant literature. Our goals were to investigate potential risk factors for stent detachment, assess how well conservative management works in such scenarios, and offer evidence to guide individualized treatment for this type of complication. The patient was a 70-year-old male who received a self-expanding Enterprise 2 stent at the origin of his left vertebral artery. At the 2-month follow-up, imaging showed stent migration and detachment into the left subclavian artery. Importantly, there was no evidence of significant vascular occlusion or acute neurological deficits. We managed the patient conservatively with dual antiplatelet and lipid-lowering therapy. After this treatment, his clinical condition improved, vital signs stayed stable, and neurological function was preserved. Our combined analysis of this case and the existing literature indicates that systemic vascular degeneration and the unique anatomical features of the vertebral artery's V1 segment are key contributors to stent detachment. In our experience, conservative treatment can lead to good outcomes for patients who are hemodynamically stable and have no ischemic symptoms. Clinical care should be tailored to each patient to avoid unnecessary repeat interventions.

## 1 Introduction

Stroke is a major contributor to morbidity and mortality in the adult population (1). The vertebral artery has slow blood flow velocity and a tortuous anatomical structure; its origin is the most common site of stenosis in the vertebrobasilar system (2). Vertebral artery origin stenosis accounts for approximately 30% of posterior circulation stroke cases. For symptomatic patients with stenosis ≥50% that remains refractory despite standardized antiplatelet therapy and whose lesions are suitable for endovascular intervention, stent placement may be considered (3). When vertebral artery stenosis occurs, the main therapeutic modalities include pharmacological treatment (primarily risk factor control and medical therapy), surgical treatment, and endovascular therapy (arterial balloon angioplasty, such as percutaneous transluminal angioplasty) (4). With the widespread clinical application of various types of stents, clinicians have identified several complications, including stent migration, malposition, displacement, incomplete deployment, and foreign body embolization. Unexpected stent detachment and embolization are among the most concerning complications leading to adverse clinical outcomes. If a malpositioned stent remains *in situ*, it may cause traumatic vascular wall injury resulting in dangerous complications such as perforation, local thrombosis, and ischemia (5–7). This article reports a case of unexpected detachment and migration of a self-expanding stent at the vertebral artery origin, which was successfully managed with pharmacological treatment.

## 2 Case presentation

The patient's baseline clinical characteristics and key time nodes of the diagnosis and treatment process are detailed in Table 1. The patient was a 70-year-old male who was admitted for cerebral infarction due to sudden onset of right-sided limb weakness lasting 12 hours. Admission cranial MRI revealed multiple acute cerebral infarcts in the left periventricular region and the genu of the corpus callosum, with stenosis of the left middle cerebral artery (MCA) M1 segment. Carotid-cerebral integrated vascular ultrasound demonstrated left vertebral artery stenosis (cervical segment: 70%–99%) with extracranial left vertebral artery pathology. The patient denied histories of hypertension, diabetes mellitus, coronary artery disease, and hyperlipidemia, as well as smoking, alcohol consumption, and family history of genetic disorders.

**Table 1:** Baseline clinical characteristics and key timeline events of the patient with left vertebral artery origin stenosis and stent detachment.

Date	Event
28 December 2023	Admitted due to sudden onset of right-sided limb weakness lasting 12 hours.
	cranial MRI revealed multiple acute cerebral infarcts in the left periventricular region and the genu of the corpus callosum, with stenosis of the left middle cerebral artery (MCA) M1 segment.
	Carotid-cerebral integrated vascular ultrasound demonstrated left vertebral artery stenosis (cervical segment: 70%–99%).
06:10 on 2 January 2024	After obtaining informed consent, cerebral digital subtraction angiography (DSA) was performed, which demonstrated dissection with severe stenosis at the origin of the left vertebral artery.
7 January	The patient was discharged with improved condition, with stable vital signs and neurological function.
24 April	Integrated cervical and cerebral ultrasound revealed severe stenosis (70–99%) of the left vertebral artery.
13 May	Admitted due to sudden onset of right-sided limb weakness, unsteady gait, and slurred speech.
	Cranial MRI shows cerebral infarction in the left cerebellar hemisphere.
08:40 on 14 May	After obtaining informed consent, cerebral angiography combined with left vertebral artery stent placement was performed.
	Preoperative DSA: Severe stenosis at the origin of the left vertebral artery (Figure 1B).
	Postoperative DSA: Patent lumen with residual stenosis <30% (Figures 2C,D).
10:10, 14 May	Postoperative vital signs were stable, and the patient was returned to the ward in stable condition.
16 May	Muscle strength recovered to grade V, with stable vital signs and neurological function.
08:00, 25 July	Readmitted due to worsening dizziness accompanied by unsteady gait.
	Cervical vascular ultrasound revealed severe stenosis at the origin of the left vertebral artery.
17:15, 25 July	After obtaining informed consent, follow-up cerebral angiography was performed.
	DSA revealed severe stenosis of the left vertebral artery at the V1 segment origin, with the stent visualized in the left subclavian artery (Figure 3A).
17:45, 25 July	Postoperative vital signs were stable, and the patient was returned to the ward.
26 July	As the stent was well-apposed to the wall of the subclavian artery without causing complete obstruction of the subclavian or vertebral artery, the condition was explained to the family. Conservative management was recommended, and following informed consent, the patient was treated with antiplatelet therapy, plaque stabilization, neurotrophic support, and rehabilitation exercises.
29 July	Vital signs were stable, neurological function was unremarkable, and the patient was discharged.
3 months after discharge	No neurological abnormalities were observed; the patient was instructed to continue medication and follow-up.

On admission, the patient's vital signs were as follows: temperature 36.4 °C, heart rate 65 beats per minute (bpm), respiratory rate 20 breaths per minute, and blood pressure 134/78 mmHg. The patient was conscious but lethargic, with slurred speech. The pupils were equal and round, with prompt light reflexes. The right nasolabial fold was flattened, and the tongue deviated to the right upon protrusion. Superficial sensation was diminished over the left extremities and trunk. Muscle strength was grade III in the right upper extremity, grade II in the right lower extremity, and grade V on the left side. Muscle tone was normal. The right Babinski sign was positive. Laboratory examinations (complete blood count, biochemistry, coagulation profile, and immunological function) revealed no significant abnormalities. After fully informing the patient's family of the condition and the proposed treatment plan and obtaining their informed consent, cerebral digital subtraction angiography (DSA) was performed. Based on preoperative imaging evaluation and the patient's clinical history, the final diagnosis was multiple cerebral infarcts, moderate left vertebral artery stenosis, and vertebral artery dissection. The patient received butylphthalide sodium chloride injection and edaravone dexborneol injection for concentrated solution to improve cerebral circulation and provide neuroprotection, aspirin enteric-coated tablets and rosuvastatin calcium tablets for antiplatelet therapy and plaque stabilization, supplemented by physical therapy and rehabilitation exercises. The patient's symptoms improved after 10 days of hospitalization, and the patient was discharged.

Four months later, the patient experienced sudden onset of right-sided limb weakness, gait instability, dizziness, and slurred speech without obvious precipitating factors, and was admitted for cerebral infarction. The patient had a prior history of cerebral infarction. On admission, vital signs were as follows: temperature 36.6 °C, heart rate 78 beats per minute (bpm), respiratory rate 20 breaths per minute, and blood pressure 117/81 mmHg. The patient was conscious with fair mental status, fluent speech, and intact cognitive function. The pupils were equal and round, with prompt light reflexes. Muscle strength was grade IV in the right upper and lower extremities and grade V on the left side. Muscle tone was normal. The right Babinski sign was positive. Laboratory examinations (complete blood count, biochemistry, coagulation profile, and immunological function) revealed no significant abnormalities. Carotid-cerebral integrated vascular ultrasound demonstrated left vertebral artery stenosis (cervical segment: 70%–99%), increased flow velocity with stenosis of the left middle cerebral artery, and extracranial left vertebral artery pathology. Cranial MRI shows cerebral infarction in the left cerebellar hemisphere. Based on preoperative imaging evaluation and the patient's clinical history, the final diagnosis was severe stenosis at the origin of the left vertebral artery.

After fully informing the patient's family of the condition and the proposed treatment plan and obtaining informed consent, the patient underwent cerebral angiography combined with left vertebral artery stent placement. Cerebral angiography revealed severe stenosis at the origin of the left vertebral artery (Figure 1B). Intraoperatively, an Enterprise2 4.0 mm × 30 mm self-expanding stent was delivered via a PLUS microcatheter. Following precise positioning, the stent was deployed. Postoperative angiography demonstrated significant improvement in vascular stenosis compared with the preoperative status, with a residual stenosis of ＜30% (Figures 2C,D). The procedure was uneventful, with stable intraoperative vital signs. Postoperative neurological examination revealed no new neurological deficit signs. To prevent in-stent thrombosis and reduce the incidence of cerebrovascular ischemic events, the patient was prescribed aspirin enteric-coated tablets, 100 mg orally once daily (qd), and clopidogrel bisulfate tablets, 75 mg orally qd, for antiplatelet therapy. The patient was discharged after 3 days of hospitalization with an improved condition. At discharge, limb muscle strength had recovered to grade V, with stable vital signs and neurological function. Upon discharge, the patient was instructed to continue regular administration of aspirin enteric-coated tablets 100 mg qd, clopidogrel bisulfate tablets 75 mg qd, and rosuvastatin calcium tablets 10 mg qd and was advised to undergo follow-up cervical vascular ultrasound or CTA of the neck 3 months later when the condition was stable.

**Figure 1:**
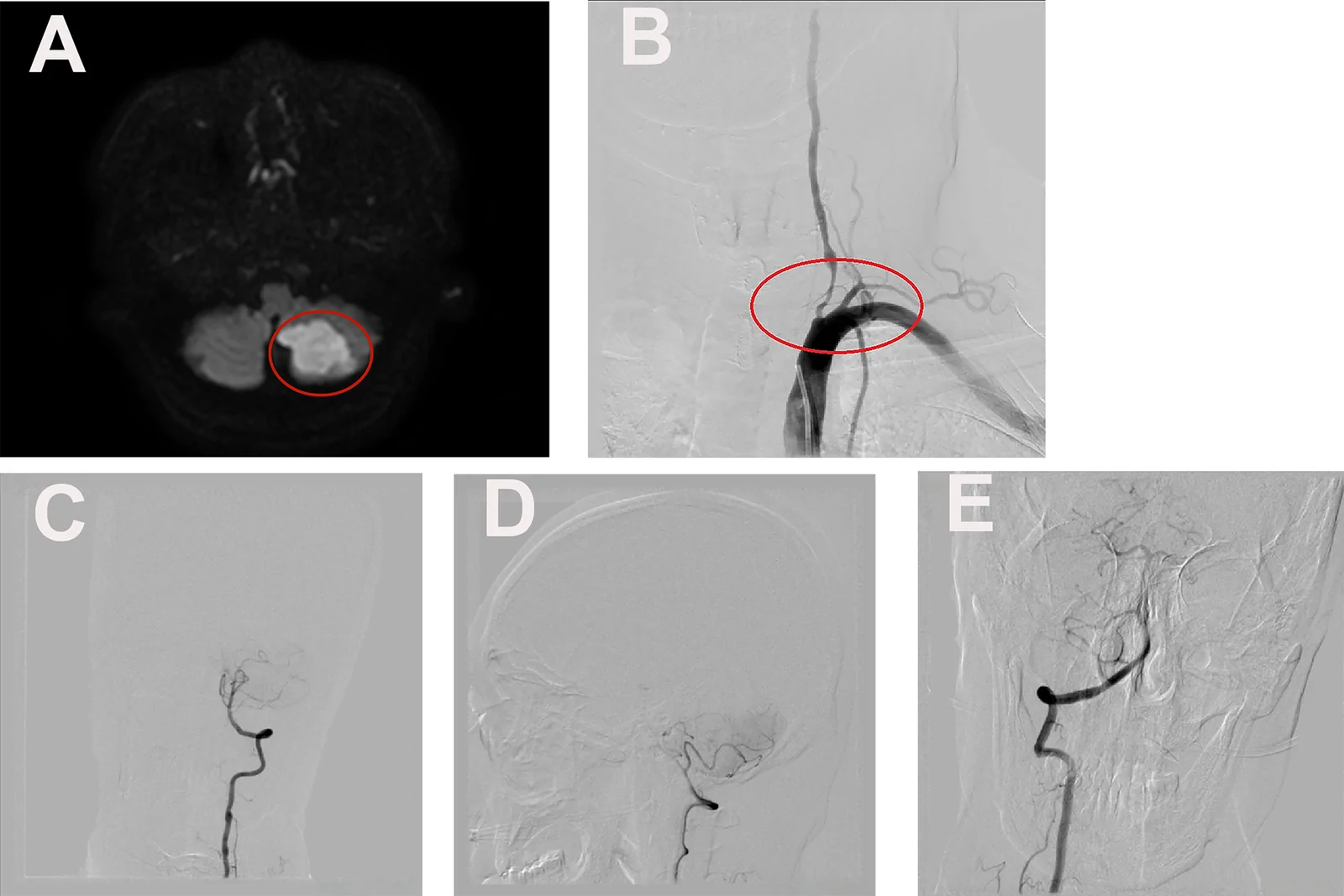
Cranial MRI: shows cerebral infarction in the left cerebellar hemisphere**.** DSA findings: **(A)** Preoperative left subclavian artery angiography showing significant focal stenosis of the vertebral artery origin, with 70%–90% stenosis; **(B)** Preoperative anteroposterior intracranial view showing clear visualization of distal branches; **(C)** Preoperative lateral intracranial view showing adequate visualization of distal branches; **(D)** Preoperative contralateral vertebral artery angiography showing patent contralateral vertebral artery with good posterior circulation collateral compensation.

**Figure 2:**
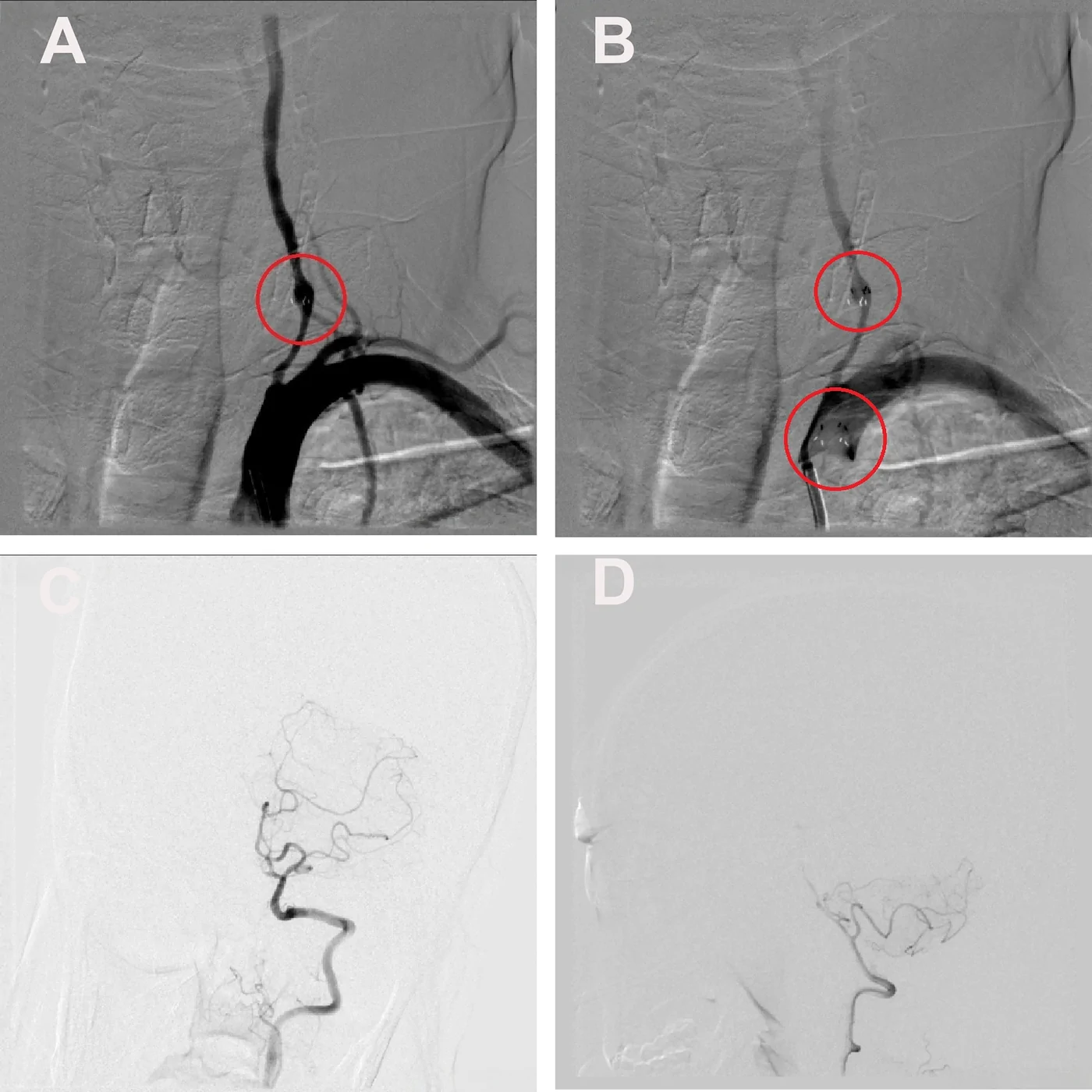
DSA results**:** postoperative angiography showed **(A)** the vertebral artery stent in place with patent vessel and good blood flow restoration; the postoperative subtraction image showed **(B)** the stent in place with patent lumen and residual stenosis <30%; the postoperative intracranial anterior-posterior view showed **(C)** and the lateral view showed **(D)** the vertebral artery stent in place with patent intracranial segment and good visualization of posterior circulation branches.

Two months postoperatively, the patient was readmitted due to worsening dizziness accompanied by unsteady gait. On admission, vital signs were stable, neurological examination revealed no positive findings, and routine laboratory tests showed no significant abnormalities. Cervical CTA demonstrated severe stenosis of the left vertebral artery V1 segment. To clarify the vascular status following stent placement, cerebral angiography was performed after informing the family and obtaining consent. The results revealed stent migration into the left subclavian artery following left vertebral artery stenting (Figure 3A). Given the patient's stable condition and absence of acute neurological deficit manifestations, a conservative treatment plan was formulated after communication with the family. The patient was prescribed aspirin enteric-coated tablets 100 mg qd, clopidogrel bisulfate tablets 75 mg qd, and rosuvastatin calcium tablets 10 mg qd orally. The patient was discharged after 4 days of hospitalization with symptom relief. At discharge, vital signs were stable with no neurological abnormalities. At the 3-month outpatient follow-up, the patient reported no recurrence of dizziness, unsteady gait, or other neurological symptoms. The patient was instructed to continue regular antiplatelet and lipid-lowering plaque-stabilizing medications, with periodic re-examination of cervical vascular imaging and neurological function, dynamic monitoring of vascular lesions and the local stent situation, and timely assessment of disease progression.

**Figure 3:**
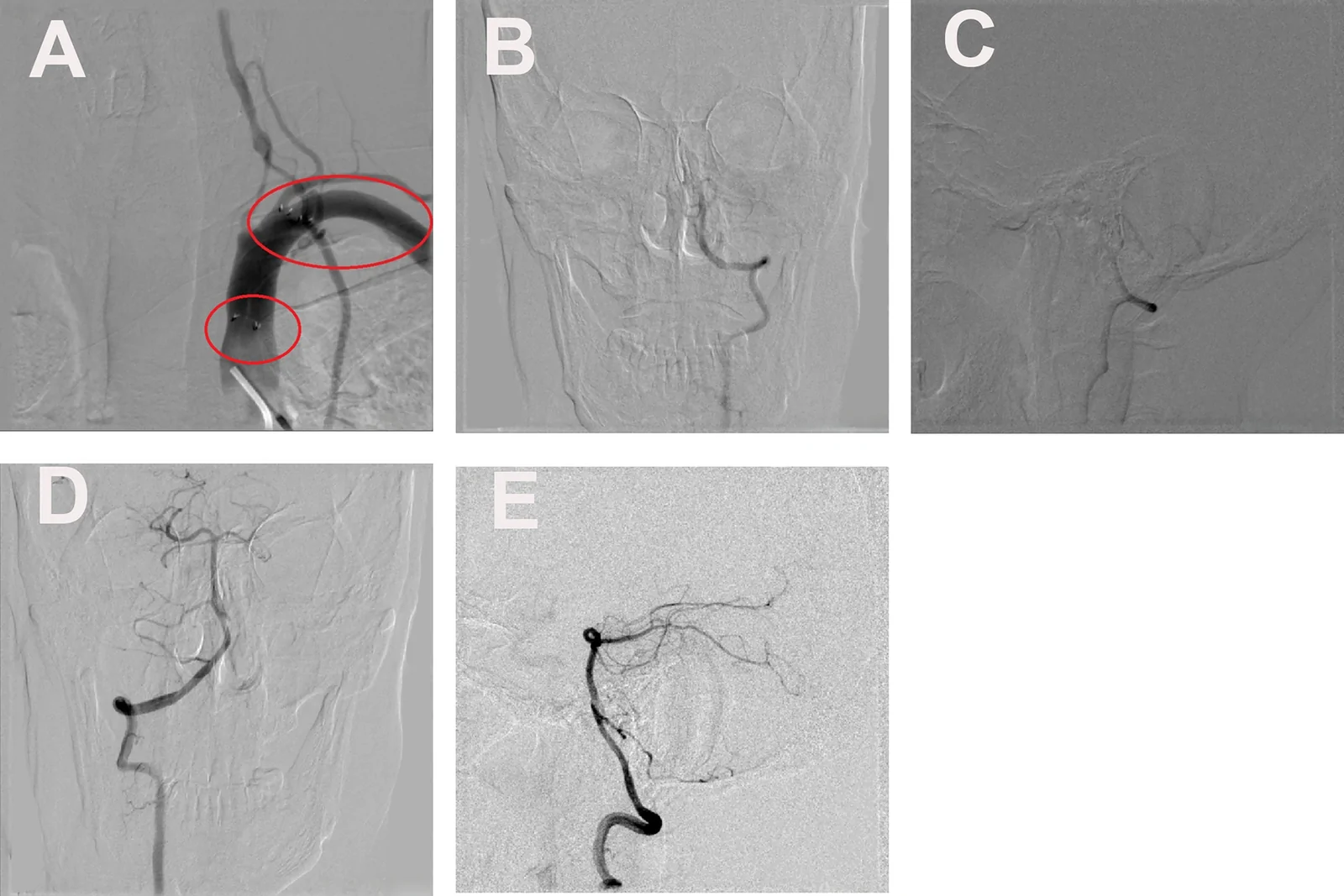
DSA results: postoperative follow-up angiography showed **(A)** the vertebral artery stent migrated to the origin of the left subclavian artery, with intact stent configuration, patent left subclavian artery blood flow, and visible opacification of the left vertebral artery without obvious signs of acute occlusion, dissection, or contrast extravasation; the distal blood flow of the left vertebral artery was patent **(B–E)** showed patent blood flow in the contralateral vertebral artery and basilar artery.

## 3 Discussion

Vertebral artery origin stenosis can lead to posterior circulation hypoperfusion, with diverse clinical manifestations ranging from mild symptoms such as dizziness and gait instability of varying degrees, to limb numbness and weakness, and posterior circulation transient ischemic attack (TIA). In severe cases, it may induce posterior circulation cerebral infarction, resulting in neurological deficit symptoms such as hemiplegia and dysphagia, or even coma and life-threatening conditions, representing one of the common etiologies of posterior circulation stroke (8). The most common causes of vertebrobasilar ischemia include embolism, penetrating small artery disease, large artery atherosclerosis, and arterial dissection (9). For patients with 50%–99% extracranial vertebral artery stenosis who continue to experience recurrent posterior circulation ischemic events despite optimal medical therapy (antiplatelet agents, lipid-lowering therapy, and risk factor control), revascularization procedures such as stent placement may be considered (10). Vertebral artery stent angioplasty effectively prevents atherosclerotic plaque detachment through stent coverage, while balloon-expandable stent deployment enables rapid restoration of the stenotic lumen to near-normal caliber. This not only improves posterior circulation perfusion but also prevents the occurrence of posterior circulation ischemic stroke, thereby enhancing patients’ quality of life. In current clinical practice, both self-expanding stents and balloon-expandable stents are commonly employed, each with distinct applicable scenarios and technical characteristics (11). However, both types of stents carry the risk of complications in clinical application, with stent migration and displacement being among the serious complications requiring particular vigilance. Stent migration may be triggered by various procedural and lesion-related factors: stent deployment under balloon negative pressure, restenting through an already deployed stent, forced advancement of stents in calcified and tortuous lesions, or withdrawal of the stent into the guiding catheter when proximal stent struts are damaged—all of which may induce this complication. Additionally, dislodgement of the guiding catheter due to tortuous vascular anatomy significantly increases the risk of stent slippage and migration (12).

Based on the clinical characteristics of this case and existing research, the causes of stent detachment in this patient can be analyzed from multiple aspects including vascular anatomical features, stent selection, technical factors, and patient-specific factors. First, the V1 segment lacks bony protection and is subject to hemodynamic impact from the subclavian artery flow, making it prone to stent detachment (13). Second, stent selection is influenced by vessel diameter, lesion location, and length. Self-expanding stents have the lowest restenosis rate (4.0%) and no periprocedural complications, offering long-term advantages; however, balloon-expandable bare metal stents and drug-eluting stents, while technically convenient, have higher restenosis rates (21.1% and 13.6%, respectively) (14). In this case, imaging measurements showed a distal vessel diameter of approximately 3.2 mm and a stenotic segment of approximately 25 mm. Given that the maximum length of balloon-expandable stents is 16 mm, which could not provide adequate coverage for this lesion, a 4.0 mm × 30 mm self-expanding stent (Enterprise2) was selected to cover the stenotic vessel proximally and distally. Third, from a technical perspective, the procedure in this case was standardized, with precise stent positioning and deployment, without inappropriate maneuvers such as balloon-negative-pressure deployment or forced stent advancement, thereby excluding technical factors as the primary cause of stent detachment. Patients undergoing vertebral artery stenting for symptomatic stenosis typically have atherosclerotic disease with plaque formation, predisposing them to vertebrobasilar hypoperfusion, with some cases complicated by calcification, dissection, or bony compression (15). This patient was an elderly male with a history of prior cerebral infarction. Preoperative examination revealed multi-site atherosclerotic plaque formation, indicating extensive degenerative changes throughout the systemic vasculature. The vascular walls exhibited significantly reduced elasticity and compliance, unable to provide adequate support and apposition for the stent.

The clinical manifestations of stent detachment lack specificity, predominantly characterized by recurrence of the original disease symptoms. In this patient, dizziness and unsteady gait recurred two months postoperatively, and cerebral angiography confirmed stent migration into the left subclavian artery. Following definitive diagnosis, timely formulation of an individualized treatment plan is crucial for improving patient prognosis. The main therapeutic approaches include device retrieval and conservative management. Device retrieval commonly employs snares, small balloons, or other devices for extraction; when retrieval fails, *in-situ* deployment or stent crushing may be performed, with surgical intervention required in rare cases. Rapid decision-making is essential to prevent complications. Conservative management, conversely, focuses primarily on pharmacological therapy supplemented by risk factor control, and is applicable when the patient's condition is stable, without obvious acute ischemic symptoms, and the site of stent detachment does not cause severe vascular obstruction.

Following definitive diagnosis of stent detachment in this patient, conservative management was selected based on the following clinical considerations: stable vital signs, absence of obvious worsening of acute neurological deficits, stent migration to the left subclavian artery with good apposition to the vessel wall, and absence of complete obstruction of the subclavian or vertebral artery. The patient was treated with dual antiplatelet therapy, lipid-lowering plaque stabilization, neurotrophic support, and circulation improvement medications. The patient's condition stabilized, with resolution of dizziness and unsteady gait, suggesting that conservative management can achieve favorable clinical outcomes in selected cases of stent detachment. A case of complete vertebral artery stent fracture with fragment migration was managed conservatively due to the patient's asymptomatic status, with long-term disease stability, suggesting that conservative management is feasible for asymptomatic patients (10). A prospective cohort study also showed that the incidence of symptomatic restenosis after vertebral artery stenting was only 2.6% (1/39), and only 25.0% of patients with stent fracture developed restenosis, suggesting that structural stent abnormalities do not necessarily lead to clinical symptoms, providing large-sample data support for conservative management of asymptomatic patients (16). In clinical practice, blind pursuit of interventional thrombectomy should be avoided, as excessive interventional manipulation may exacerbate vascular endothelial injury and increase the risks of thrombosis and vascular perforation; therefore, the indications for interventional therapy must be strictly adhered to.

## 4 Conclusion

Stent placement is an effective means of improving posterior circulation perfusion in patients with vertebral artery origin stenosis. However, due to the influence of vascular anatomical characteristics and degenerative vascular changes, there exists a potential risk of stent detachment. The clinical manifestations of stent detachment lack specificity, predominantly presenting as recurrence of the original disease symptoms. The key to definitive diagnosis lies in cerebral angiography.

The clinical management of stent detachment requires individualized adjustment based on the patient's actual condition. For patients with stable vital signs, without severe vascular occlusion or worsening acute neurological deficits, conservative management with dual antiplatelet therapy, lipid-lowering plaque stabilization, and neurotrophic support—following the treatment strategy employed in this case—can achieve favorable clinical outcomes while avoiding additional vascular intimal injury from secondary interventional procedures. The successful control of this patient's condition through conservative management provides practical reference for the management of similar stent detachment cases in clinical practice. Given that relevant research data on such complications remain limited, large-scale clinical studies are still needed to further identify high-risk factors for stent detachment and to explore more standardized and efficient individualized treatment protocols.

## Data Availability

The original contributions presented in the study are included in the article/Supplementary Material, further inquiries can be directed to the corresponding author.
